# Impact of Biologics on Oscillometry Defined Small Airway Dysfunction in Uncontrolled Asthma

**DOI:** 10.1111/cea.70349

**Published:** 2026-05-24

**Authors:** Robert Greig, Philipp Suter, Rory Chan, Brian Lipworth

**Affiliations:** ^1^ Scottish Centre for Respiratory Research University of Dundee Dundee UK

**Keywords:** asthma, benralizumab, biologics, dupilulmab, mepolizumab, monoclonal antibodies, oscillometry, small airways dysfunction, tezepelumab

## Abstract

Small airways dysfunction (SAD) is a recognised treatable trait within severe asthma, associated with worse symptom control and increased exacerbations. It can be measured using forced oscillometry technique (FOT). FOT is an effective and effort‐independent method of measuring peripheral lung resistance and compliance. Type 2 inflammation can affect the small airways via eosinophilic inflammation, mucus plugging and smooth muscle inflammation. These are all potential targets of monoclonal antibody therapy. Clinical trials have shown that mepolizumab, dupilumab and tezepelumab all effect both significant and clinically meaningful improvements in oscillometry‐defined SAD; however, benralizumab does not. Furthermore, in indirect matched head‐to‐head comparisons, dupilumab exerts greater improvements compared to either benralizumab or tezepelumab. Future research should consider oscillometry‐defined SAD being incorporated as a key clinical outcome in phase 2 studies as new monoclonal antibodies are developed, such as bispecifics, especially both dual upstream (IL33/TSLP) or downstream blockade (IL4/5/13).

## Key Messages

- Oscillometry defined small airways dysfunction is a recognised treatable trait in severe asthma.
- Dupilumab, mepolizumab and tezepelumab but not benralizumab resulted in significant improvements in oscillometry defined SAD.
- Differing effects in SAD improvements may be present between upstream and downstream type 2 blockade.

## Graphical abstract

Forced oscillometry technique defined small airways dysfunction as a treatable trait in asthma. Monoclonal antibodies can improve small airways dysfunction, but their effects vary by mechanism of action.

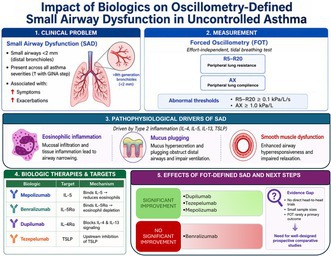

The small airways refer to the bronchioles beyond the 8th generation, measuring under 2 mm in diameter. Small airways dysfunction (SAD) has been shown to be an independent determinant for poor symptom control and exacerbation frequency in asthma [1]. The ATLANTIS study showed that SAD can be present in patients with asthma at all GINA steps with an increasing prevalence as severity escalates [2].

There are various methods by which clinicians can measure small airways function. One commonly used method is using spirometry to measure volume dependent airway closure as the forced expiratory flow rate between 25% and 75% of forced vital capacity (FEF_25–75_). However, this is highly variable and declines rapidly with age [3]. Other methods include body plethysmography, using the ratio of residual volume to total lung capacity or total airways resistance; however, this assessment method is not specific for the small airways. Another technique is multiple breath nitrogen washout to assess acinar ventilation heterogeneity, which is highly sensitive but difficult to perform and requires specialist equipment and is therefore limited to research [4].

Forced oscillometry technique (FOT) is an easy to use, effort‐independent method of assessing the small airways either with impulses or continuous sound or air waves emitted from a loudspeaker or vibrating mesh, which are superimposed upon normal tidal breathing at various frequencies, allowing for a pressure/flow loop of the respiratory impedance to be measured. From this impedance, the resistance (R) and reactance (X) are calculated. R at 5 Hz (R5) represents resistance in the whole airway and R at 20 Hz (R20) in the large airways. Thus, the difference between R5 and R20 (R5‐R20) reflects heterogeneity of peripheral lung resistance within small airways. AX is the area under the reactance curve between 5 Hz and resonant frequency representing peripheral lung compliance [5]. Previous studies have shown that an R5‐R20 with a value of ≥ 0.1 kPa/L/s and/or an AX of ≥ 1.0 represent clinically abnormal peripheral lung resistance and compliance, respectively, being predictive of poorer asthma control and increased exacerbations [6]. The minimal clinical important difference (MCID) values for R5‐R20 and AX have been proposed to be ≥ 0.06 kPa/L/s and ≥ 0.65 kPa/L respectively although this was in a heterogeneous group of asthma patients according to severity [7]. In severe asthma patients, the biological variability values corresponding to a change in ACQ score of 0.6 were ≥ 0.04 kPa/L/s and ≥ 0.39 kPa/L [8]. FOT has also been shown to be a more sensitive marker when compared to spirometry in assessing bronchodilator response [9]. FOT is therefore an easy and efficient method of evaluating the small airways.

The introduction of monoclonal antibodies has revolutionised the treatment of severe uncontrolled asthma. These medications have systemic effects and thus are able to penetrate these distal airways. Previously, this was achieved through either systemic corticosteroids or extra‐fine particle inhaled therapies with particles smaller than 2 μm [10] which precludes the bulk of currently available inhaled therapies. The monoclonal antibodies act on various aspects of the type 2 inflammatory cascade. Currently, the majority of the monoclonal antibodies target the downstream cytokines such as interleukin (IL) 4, IL‐5 and IL‐13 with tezepelumab acting upstream on the epithelial alarmin thymic stromal lymphopoietin (TSLP). All the monoclonal antibodies discussed within this article have been shown to significantly reduce exacerbations and improve symptom control [11, 12, 13, 14].

Here we review the evidence base for the effects of monoclonal antibodies on oscillometry defined SAD (FOT‐SAD). A literature review was undertaken to identify both prospective and real‐life studies reviewing the effects of monoclonal antibodies on oscillometry. Both PubMed and Google Scholar were searched using the terms oscillometry, small airway dysfunction, biologics, monoclonal antibodies, benralizumab, mepolizumab, dupilumab, omalizumab, tezepelumab and reslizumab. Only studies that reviewed individually named monoclonal antibodies were considered; combined cohorts, congress abstracts and case reports were excluded. Neither omalizumab nor reslizumab have published data on FOT‐SAD and as such are not discussed within this manuscript. All studies reviewed patients with severe uncontrolled asthma with the exception of Diver et al. [14] and Washko et al. [15] which reviewed moderate to severe uncontrolled asthma cohorts (Table 1).

**TABLE 1:** Summary of the clinical trials reporting oscillometry defined small airways dysfunction.

Biologic	Manuscript (reference)	*N*	Duration (months)	Mean baseline R5‐R20 (kPa/L/s)	Mean change (95% CI) in R5‐R20 (kPa/L/s)	Mean baseline AX (kPa/L)	Mean change (95% CI) in AX (kPa/L)
Mepolizumab	Kotsiou 2025 [16]	40	6	0.12	−0.09 (−0.12, −0.06)	1.2	−0.6 (−0.89, −0.31)
	Bonini 2025 [17]	16	12	0.25	−0.06 (−0.12, −0.01)	NR	NR
Benralizumab	Chan 2023 [18]	21	3	0.14	0.00 (−0.04, 0.04)	2.77	−0.46 (−1.43, 0.50)
	Chan 2023 [19]	21	8	0.24	−0.01 (−0.06, 0.04)	3.55	−0.40 (−1.09, 0.30)
	Shirai 2020 [20]	19	6	0.07^a^	0.005 (−0.18, 0.08)^a^	0.25^a^	0.03 (−1.99, 1.45)^a^
Dupilumab	Stewart 2025 [21]	24	3	0.13	−0.058 (−0.094, −0.022)	2.59	−1.26 (−2.18, −0.34)
	Washko 2025 [15]	72	6	0.17	−0.05 (−0.07, −0.03)	3.43	−0.76 (−1.28, −0.08)
	Chan 2023 [22]	16	4.5	0.21	−0.12 (−0.21, −0.04)	3.03	−2.24 (−3.82, −0.65)
Tezepelumab	Diver 2021 [14]	54	7–12	0.13	0.01 (−0.04, 0.06)	2.22	0.32 (−0.54, 1.18)
	Greig 2025 [23]	15	7	0.32	−0.17 (−0.29, −0.06)	3.99	−2.24 (−4.09, −0.73)
	Menzella 2025 [24]	17	6	0.12	−0.02 (−0.17, 0.13)	NR	NR
	Kupershmidt 2026 [25]	34	6	0.16^a^	0.10 (−0.11, 0.30)	1.62^a^	1.10 (−0.72, 5.26)

Abbreviation: NR, not reported.

^a^ Median.

## 1 Potential Targets Within Small Airway Dysfunction

**FIGURE 1:**
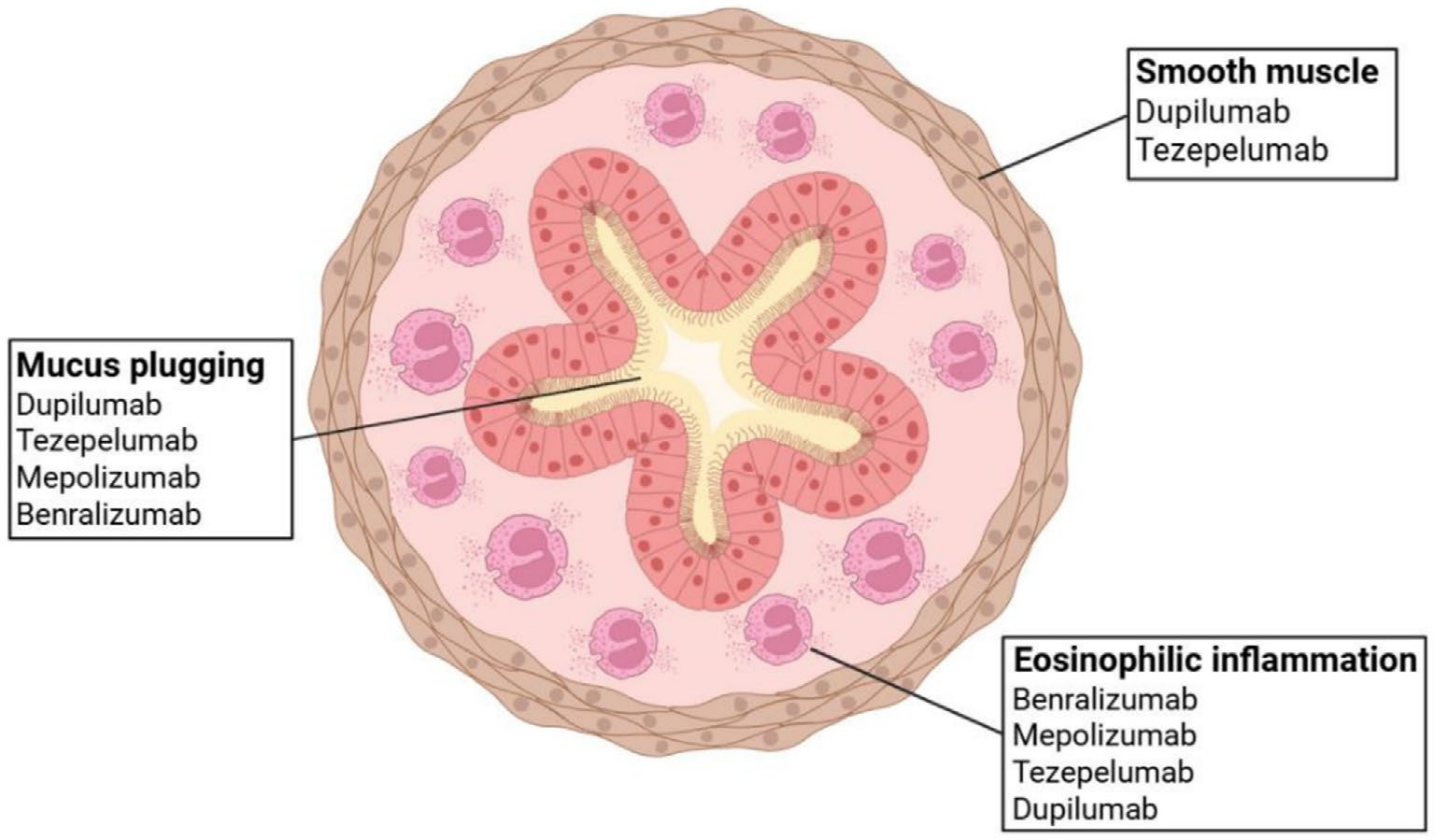
The putative mechanisms for the effects of monoclonal antibodies on small airway function.

There are three key putative modes of action by which monoclonal antibodies may improve SAD: eosinophilic inflammation, mucus plugging and smooth muscle inflammation (Figure 1) [10].

Eosinophils can be measured in the blood, sputum, mucosa and submucosa of the airways. By attenuating eosinophilic mucosal infiltration, monoclonal antibodies may improve luminal airway calibre in peripheral airways [26].

Mucus plugging has been shown to correlate with FeNO, serum eosinophils, serum IL‐13 along with both serum and bronchoalveolar lavage IL‐5 and eosinophil‐derived neurotoxin, a biomarker of eosinophilic degranulation [27]. This illustrates the multifaceted effects of type 2 inflammation on mucus plugging including the effects of eosinophil cytotoxicity by IL‐5, goblet cell hyperplasia and increased MUC5AC production driven by IL‐13. Mucus plugging can be evaluated using high resolution CT imaging. In this approach, the 10 major bronchopulmonary segments of each lung are assessed for the presence of mucus occlusions. One point is given for each segment which contains a plug, resulting in a mucus plug score (MPS) ranging from 0 to 20 [28]. Improvements in MPS have been shown to correlate closely with airway calibre and symptom scores [29]. By dissolving mucus plugs with monoclonal antibodies, distal lung ventilation and associated SAD can be improved.

A study reviewing the effects of the type 2 cytokines on airway smooth muscle cells in vitro found that IL‐4 and IL‐13 but not IL‐5 accentuated the AHR response to histamine and leukotriene D4 [30]. Thus, one could hypothesise that monoclonal antibodies which exert their effects via IL‐4 or IL‐13 will be able to meaningfully improve SAD via airway smooth muscle relaxation. Indeed, when the change of FEF_25–75_ in response to tezepelumab, expressed as both absolute change and corrected for volume (FEF_25–75_/FVC), is compared with change in FeNO and eosinophils as surrogates for IL‐13 and IL‐5 respectively, change in FeNO but not eosinophils change was significantly correlated in keeping with the in vitro findings [31].

Reviewing these mechanisms, one could hypothesise that all the currently available monoclonal antibodies could exert a beneficiary effect on FOT‐SAD.

## 2 Mepolizumab and Benralizumab

Both mepolizumab and benralizumab achieve their effects via the IL‐5 pathway. Mepolizumab is a humanised IgG1k monoclonal antibody, which selectively binds with high affinity to IL‐5. This in turn inhibits the interaction of IL‐5 with its receptor unit which consists of two subunits: IL‐5‐specific α subunit (IL‐5Rα) and a non‐specific βc chain [32]. Via this mechanism, mepolizumab acts on all cells which carry the IL‐5 receptor including eosinophils, basophils and mast cells. Benralizumab is also a humanised IgG1k monoclonal antibody that specifically binds to IL‐5Rα. This in turn results in two distinct processes: the blockage of IL‐5 and its receptor, alongside the recruitment of natural killer cells by binding to the Fc*γ*IIIRa receptor, thereby inducing apoptosis of eosinophils [33]. Mepolizumab and benralizumab significantly reduce eosinophilia with both being shown to reduce the terminal differentiation of eosinophils within the bone marrow although only benralizumab resulted in complete suppression [34, 35]. Benralizumab has been shown to be superior at suppressing blood eosinophilia, exerting a rapid depletion of serum eosinophils to nearly undetectable levels within 24 h which is sustained over 30 days [36]. However, eosinophil detection in the sputum persists despite the complete suppression of eosinophils in the bone marrow and the blood [35]. Both mepolizumab and benralizumab have also been shown to reduce MPS [37, 38].

**FIGURE 2:**
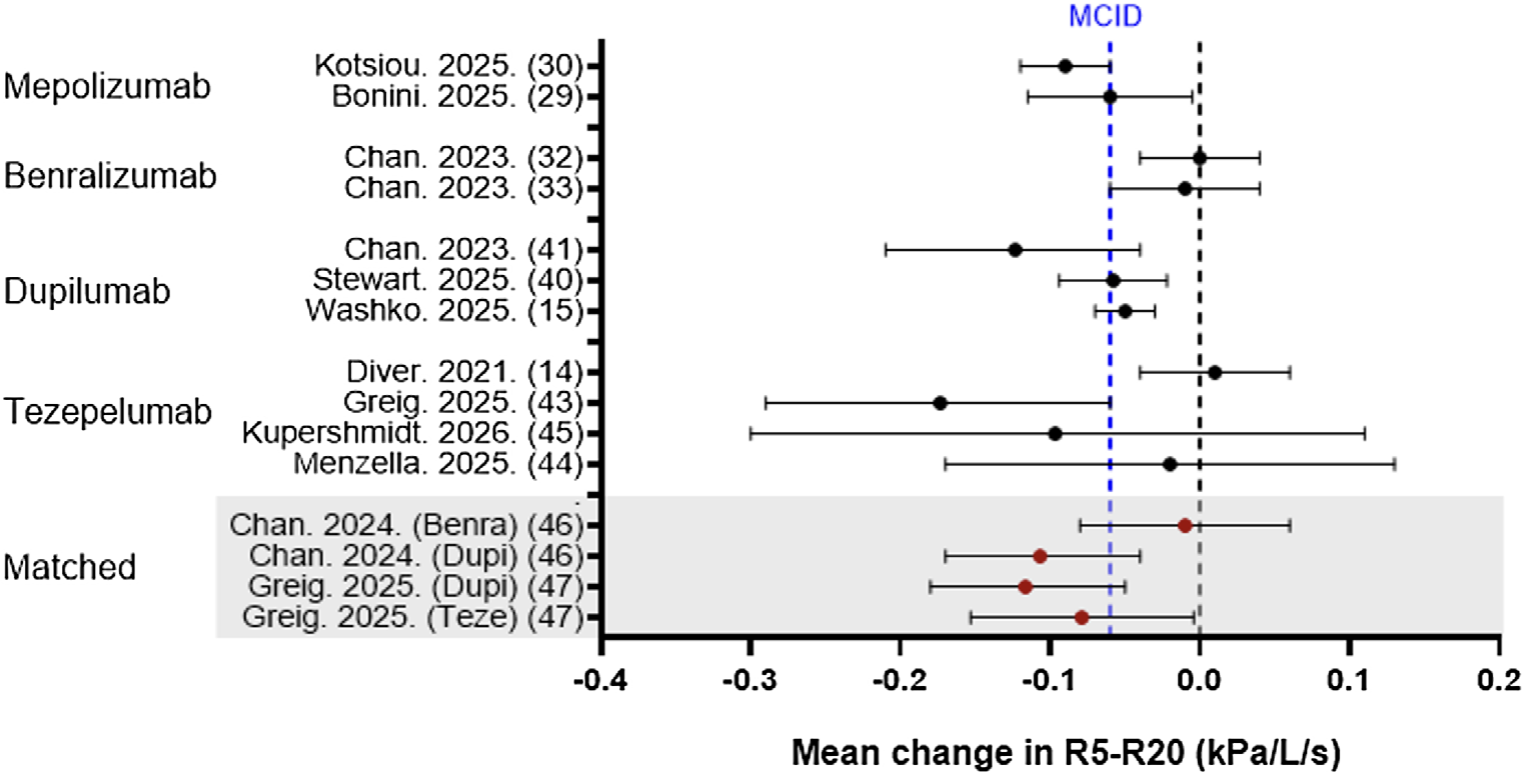
Comparison of the mean change in R5‐R20 (95% CI) between the studies including an indirectly matched sub‐analysis of participants from Chan and Greig (red). 95% CI which exclude zero denotes a significant difference as change from baseline.

**FIGURE 3:**
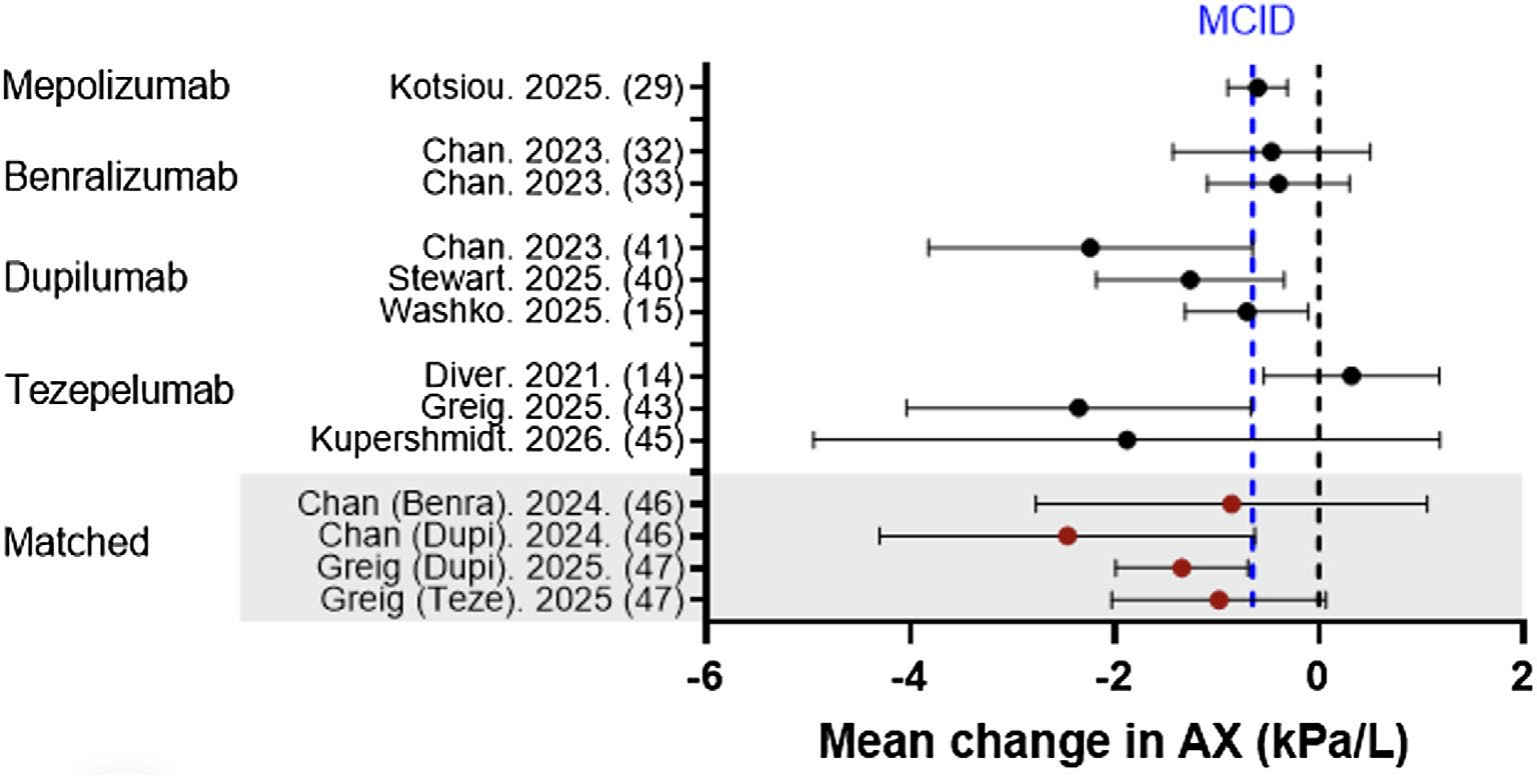
Comparison of the mean change in AX (95% CI) between the studies including an indirectly matched sub‐analysis of participants from Chan and Greig (red). 95% CI which exclude zero denotes a significant difference as change from baseline.

Regarding reporting the effects on FOT‐SAD, there are two prospective studies which review the effects of mepolizumab [16, 17] and two for benralizumab [18, 20] with a further retrospective study in benralizumab [19]. By reviewing the forest plots (Figures 2 and 3), mepolizumab, but not benralizumab, results in significant improvements in both R5‐R20 and AX.

The IMPOSE study by Kotsiou et al. [16] prospectively analysed the effects of mepolizumab on respiratory mechanics using IOS. They compared 40 patients with severe asthma who were started on mepolizumab to both patients with well controlled asthma (*n* = 40) and healthy controls (*n* = 50) over a 6 month period. Within the severe asthma group, mean values of R5‐R20 and AX improved significantly from 0.12 kPa/L/s to 0.03 kPa/L/s and 1.20 kPa/L to 0.60 kPa/L, respectively; however, only the mean change for R5‐R20 exceeded the MCID of 0.06 kPa/L/s whereas both exceeded the severe asthma biological variability values of 0.04 kPa/L/s and 0.39 kPa/L. The change in R5‐R20 and AX were both significant when compared to the healthy control (mean difference: 0.07 kPa/L/s and 0.07 kPa/L respectively). A small prospective, real life study found similar improvements over 1 year in a cohort of 16 patients with 81% having FOT‐SAD at baseline. The total mean reduction in R5‐R20 was 0.06 kPa/L/s which was both statistically significant and exceeding the biological variability value. However, AX was not reported in this study [17].

A prospective single‐arm study of 19 patients evaluated the effects of benralizumab on FOT‐SAD as the primary outcome over 6 months. This study found no significant improvements in either R5‐R20 or AX [20]. Another prospective study (*n* = 21) assessing the effects of benralizumab on airway hyper‐responsive (AHR) as the primary outcome also did not show any significant improvements for the mean change in either R5‐R20 (0.00 kPa/L) or AX (−0.46 kPa/L) despite significant improvements in mannitol AHR exceeding the MCID [18]. Furthermore, a real‐life retrospective study of 21 patients over 8 months identified patients with FOT‐SAD at baseline with a mean baseline R5‐R20 of 0.21 kPa/L/s. Despite significant improvements in FEV_1_ and ACQ both greater than the respective MCIDs, no significant improvement in either R5‐R20 or AX was observed [19].

## 3 Dupilumab

Dupilumab is an IgG4 humanised monoclonal antibody. It exerts its action through binding to the IL‐4Rα subunit which is shared by both the IL‐4 (type 1) and IL‐13 (type 2) receptor complexes. This in turn means that dupilumab accomplishes a dual blockade of both IL‐4 and IL‐13 signalling. Type 1 and type 2 receptor complexes are present throughout the type 2 inflammatory cascade with type 1 complexes present on B cells, eosinophils, fibroblasts, monocytes, T_H_0 cells and T_H_2 cells; while type 2 complexes are found in activated B cells, eosinophils, epithelial cells, fibroblasts, goblet cells, monocytes and smooth muscle cells [39, 40]. Significant improvements in multiple type 2 mediated conditions including asthma, atopic dermatitis, eosinophilic oesophagitis, chronic rhinosinusitis with nasal polyposis have been seen with dupilumab [13, 41, 42]. Dupilumab effects on eosinophils are the inverse of the other monoclonal antibodies with a transient increase of blood eosinophils by blocking the eosinophil transmigration into tissue. This has been noted in dupilumab trials when compared to the placebo with blood levels usually returning to their baseline or lower after 24 weeks [43]. Interestingly, the overall benefits from dupilumab were greater if a patient's baseline blood eosinophil count was ≥ 300cells/μL. In terms of mucus plugging, the VESTIGE study showed significant improvements [44].

We identified three papers which report FOT outcomes although this was not the primary outcome for either of the two prospective studies [15, 21] with only one retrospective real life study focusing on a FOT‐SAD defined cohort [22]. Figures 2 and 3 show that dupilumab consistently resulted in significant improvements in both R5‐R20 and AX.

A post hoc review of the VESTIGE study reviewed 72 type 2 high, moderate to severe patients receiving dupilumab and 37 placebo over 6 months. Here FOT was a secondary end point. The dupilumab cohort, although not selected for FOT‐SAD at baseline, exhibited a raised mean R5‐R20 of 0.17 kPa/L/s and AX of 3.43 kPa/L and experienced a significant mean improvement in response within the dupilumab arm amounting to −0.05 kPa/L/s and −0.76 kPa/L, respectively. When compared to placebo, these improvements remained significant for both R5‐R20 and AX with respective mean difference of −0.06 kPa/L/s and −1.43 kPa/L, while responder analysis using biological variability values showed that patients were 73% and 57% more likely to respond to dupilumab than placebo for R5‐20 and AX, respectively [15]. Similar results were found by Stewart et al. in another post hoc analysis of a prospective single arm study reviewing the effects of dupilumab on AHR as the primary outcome in 23 type 2 high severe asthma patients. Significant mean improvements in R5‐R20 of −0.06 kPa/L/s and in AX of −1.26 kPa/L were identified with responder analysis revealing 42% and 50% of patients exceeding the respective biological variability values [21]. Here the response may have been blunted as patients also tapered their inhaled corticosteroid dose during the study period. Unlike these prospective studies which did not select their patients based on their baseline FOT‐SAD, Chan et al. reviewed a real life cohort of 16 patients who all exhibited FOT‐SAD at baseline. In this cohort, dupilumab resulted in a significant improvement in both R5‐R20 and AX with 75% of the cohort improving both values by greater than the biological variability values [22].

## 4 Tezepelumab

Tezepelumab is an IgG2 humanised monoclonal antibody which binds to TSLP and therefore inhibits the binding of TSLP to TSLP receptors. While the downstream mechanism of action is not fully confirmed, TSLP receptors are found on both type 2 and non‐type 2 inflammatory cells including eosinophils, basophils, mast cells, airway smooth muscle cells, group 2 innate lymphoid cells, lymphocytes, dendritic cells and monocytes/macrophages [45]. This in turn has diminishing effects on downstream cytokines including IL‐4, IL‐5 and Il‐13. Thus, significant reductions in eosinophils are seen in both blood and the mucosa, although not as complete when compared to mepolizumab or benralizumab [14]. Tezepelumab has also been shown to reduce the MPS in a post hoc review of the CASCADE trial [27].

The effects of tezepelumab on FOT were first analysed in the prospective placebo controlled study CASCADE as an exploratory outcome. No improvement was seen in R5‐R20 or AX in response to tezepelumab, despite these patients having FOT‐SAD at baseline (mean R5‐R20: 0.13 kPa/L/s; AX 2.22 kPa/L). Additionally, against placebo, the mean change from baseline was 0.03 kPa/L/s for R5‐R20 and 0.56 kPa/L for AX in favour of the placebo [14]. Three further studies have reviewed the effect of tezepelumab on FOT values in real‐life cohorts. One retrospective study identified 15 patients who all exhibited baseline FOT‐SAD and had significant improvements in both R5‐R20 and AX with the lower 95% CI for R5‐R20 being equal to the MCID and the 95% CI for AX exceeding it, with 87% and 73% of patients exceeding the biological variability value respectively [23]. The other two prospective real life studies, Menzella et al. [24] and Kupershmidt et al. [25] did not select patients on FOT‐SAD but rather identified them as a subgroup, with 7/17 patients (41%) and 26/34 patients (76%), respectively. Menzella et al. defined FOT‐SAD as a reactance > 150% predicted (Xrs). This limits the comparison with the other studies, given they report R5‐R20 or AX as the key outcomes. This difference is further highlighted, given at baseline the Menzella et al. cohort does not have a significant difference between R5‐R20 when comparing the SAD versus no SAD subgroups (*p* = 0.17). Kupershmidt et al. reported abnormal median baseline R5‐R20 and AX of 0.16 kPa/L/s and 1.62 kPa/L, respectively. The cohort experienced a significant mean improvement greater than the MCID in both R5‐R20 and AX at 0.10 kPa/L/s and 1.10 kPa/L, respectively, however, the 95% CI were wide. Of the total cohort, 76% of patients initially had FOT‐SAD, which reduced to 44% post tezepelumab [25]. The forest plots of these studies (Figures 2 and 3) show that in response to tezepelumab, the change in R5‐R20 and AX was much more variable when compared to dupilumab; however, there remains an overall beneficial impact on FOT‐SAD.

## 5 Comparative Studies

There are limited data regarding head‐to‐head comparisons between monoclonal antibodies regarding SAD and comparisons between the aforementioned studies are difficult given the varying FOT‐SAD baselines. To date, there are two studies which matched patients based on a baseline R5‐R20 ≥ 0.10 kPa/L/s with the aim to compare benralizumab versus dupilumab [46] and dupilumab versus tezepelumab [47]. The former identified patients from two prospective phase 4 clinical trials with equal duration of follow‐up and high dose ICS at run‐in [46]. The latter was a real‐life study where patients were not only matched on the pre‐monoclonal antibody baseline R5‐R20 but also the ICS dose, duration of follow‐up and BMI [47]. Unsurprisingly, both studies showed that both dupilumab and tezepelumab significantly improved FOT‐SAD whereas benralizumab did not, in keeping with the literature. On reviewing the improvement as relative % improvement, dupilumab exerted significantly greater improvements on FOT‐SAD compared to both benralizumab (R5‐R20: 44.2% (2.1, 86.3) and AX: 42.6% (1.7, 83.5)) and tezepelumab (R5‐R20: 18.52% (3.97, 33.07) and AX: 26.23% (6.92, 45.55)).

## 6 Further Considerations

On reviewing the effects of the various monoclonal antibodies in relation to FOT‐SAD, two key questions stand out. Despite both targeting the IL‐5 pathway, mepolizumab, but not benralizumab, has been shown to influence FOT‐SAD, despite near complete eosinophil depletion with the latter. One may consider that this could be due to the effect of mepolizumab on the other type 2 inflammatory cells which express the IL‐5 receptor, such as mast cells and basophils. The other is the variability of response of tezepelumab when compared to dupilumab between respective trials and in the indirect head‐to‐head. By having its action downstream, dupilumab blocks the IL‐4Rα subunit and thus IL‐4 and IL‐13. Since the effects of tezepelumab are upstream via TSLP, one may consider the presence of putative escape of other upstream alarmins, such as IL‐33, which stimulates downstream release of downstream IL‐4, IL‐5, and IL‐13. In this regard, IL‐33 directly activates macrophages, mast cells, basophils, eosinophils, and innate lymphoid cells, which are all key components within the type 2 inflammatory cascade [48] and so incomplete upstream alarmin blockade may not be as effective when compared to downstream in attenuating FOT‐SAD.

Another factor that must also be considered is the starting baseline values of FOT. If the patient cohort does not have FOT‐SAD, then there may be no room for potential improvement. This is demonstrated in both the dupilumab and tezepelumab cohorts [46, 47] where selected subgroups with FOT‐SAD at baseline were able to show relatively greater improvements. Current evidence is limited by small sample sizes and the lack of studies using oscillometry as a primary outcome.

FOT‐SAD has been shown to be associated with a higher symptom burden and increased exacerbations [1, 49]. Thus, one may consider selecting a monoclonal antibody that demonstrates efficacy in FOT‐SAD rather than one that does not or lacks supporting evidence in patients with FOT‐SAD. However, given the lack of direct head‐to‐head trials, definitive conclusions are limited. This highlights the need for further research to provide clinicians with the information required to make robust and informed decisions regarding monoclonal antibody selection in this cohort of patients.

## 7 Conclusions

FOT‐SAD is increasingly recognised in patients with uncontrolled asthma as a potential treatable trait, albeit effects vary considerably between monoclonal antibodies. Dupilumab, mepolizumab and tezepelumab, but not benralizumab, resulted in significant improvements in oscillometry defined SAD. As new monoclonal antibodies are developed, such as bispecifics, especially both dual upstream (IL33/TSLP) or downstream blockade (IL4/5/13), consideration should be given towards FOT‐SAD being incorporated as a key clinical outcome in phase 2 studies.

## Author Contributions

R.G.: Conceptualisation, data curation, formal analysis, visualisation, writing – original draft. P.S.: Writing – reviewing and editing and visualisation. R.C.: Writing – reviewing and editing. B.L.: Conceptualisation, writing – reviewing and editing and supervision.

## Funding

The authors have nothing to report.

## Conflicts of Interest

Dr. Greig reports personal fees (talks) from AstraZeneca and institutional grants (equipment) from Chiesi. Dr. Suter reports personal fees (talks) from AstraZeneca, personal fees (talks) from GSK, grants from Lung League Fribourg (Switzerland), grants from Swiss Lung Foundation (Switzerland). Dr. Chan reports institutional grants awarded from Asthma+Lung UK, Chiesi, AstraZeneca and GSK; serving on advisory boards for AstraZeneca and Vitalograph; personal fees (talks and/or drafting educational material) from AstraZeneca, Chiesi, Thorasys and Vitalograph; and support attending meetings from AstraZeneca, Chiesi, NIOX, Sanofi‐Regeneron and Vitalograph. Dr. Lipworth reports non‐financial support (equipment) from GSK; grants, personal fees (consulting, talks and advisory board), other support (attending ATS and ERS) and from AstraZeneca; personal fees (talks and consulting) from Sanofi, personal fees (consulting, talks and advisory board) from Circassia in relation to the submitted work; grants, personal fees (consulting, talks, advisory board), other support (attending ERS) from Teva, personal fees (talks and consulting), grants and other support (attending ERS and BTS) from Chiesi, personal fees (consulting) from Lupin, personal fees (consulting) from Glenmark, personal fees (consulting) from Dr. Reddy, personal fees (consulting) from Sandoz; grants, personal fees (consulting, talks, advisory board), other support (attending BTS) from Boehringer Ingelheim, grants and personal fees (advisory board and talks) from Mylan outside of the submitted work; and the son of BJL is presently an employee of AstraZeneca.

## Data Availability

Data sharing not applicable to this article as no datasets were generated or analysed during the current study.
